# Chronic Critical Illness Elicits a Unique Circulating Leukocyte Transcriptome in Sepsis Survivors

**DOI:** 10.3390/jcm10153211

**Published:** 2021-07-21

**Authors:** Dijoia B. Darden, Gabriela L. Ghita, Zhongkai Wang, Julie A. Stortz, Maria-Cecilia Lopez, Michael C. Cox, Russell B. Hawkins, Jaimar C. Rincon, Lauren S. Kelly, Brittany P. Fenner, Tezcan Ozrazgat-Baslanti, Christiaan Leeuwenburgh, Azra Bihorac, Tyler J. Loftus, Frederick A. Moore, Scott C. Brakenridge, Henry V. Baker, Rhonda Bacher, Alicia M. Mohr, Lyle L. Moldawer, Philip A. Efron

**Affiliations:** 1 Department of Surgery, University of Florida College of Medicine, Gainesville, FL 32610, USA; Dijoia.Darden@surgery.ufl.edu (D.B.D.); Julie.Stortz@surgery.ufl.edu (J.A.S.); Michael.Cox@surgery.ufl.edu (M.C.C.); Russell.Hawkins@surgery.ufl.edu (R.B.H.); Jaimar.Rincon@surgery.ufl.edu (J.C.R.); Lauren.Kelly@surgery.ufl.edu (L.S.K.); Brittany.Fenner@surgery.ufl.edu (B.P.F.); Tyler.Loftus@surgery.ufl.edu (T.J.L.); Frederick.Moore@surgery.ufl.edu (F.A.M.); Scott.Brakenridge@surgery.ufl.edu (S.C.B.); Alicia.Mohr@surgery.ufl.edu (A.M.M.); Lyle.Moldawer@surgery.ufl.edu (L.L.M.); 2 Department of Biostatistics, University of Florida, Gainesville, FL 32610, USA; glghita0429@ufl.edu (G.L.G.); zkwang@ufl.edu (Z.W.); rbacher@ufl.edu (R.B.); 3 Department of Molecular Genetics and Microbiology, University of Florida, Gainesville, FL 32610, USA; mclopez@UFL.EDU (M.-C.L.); hvbaker@UFL.EDU (H.V.B.); 4 Department of Aging and Geriatric Research, University of Florida, Gainesville, FL 32610, USA; Tezcan.OzrazgatBaslanti@medicine.ufl.edu (T.O.-B.); cleeuwen@ufl.edu (C.L.); 5 Department of Medicine, University of Florida College of Medicine, Gainesville, FL 32610, USA; abihorac@ufl.edu

**Keywords:** human, sepsis, RNA, transcriptome, leukocytes, immunology

## Abstract

Surgical sepsis has evolved into two major subpopulations: patients who rapidly recover, and those who develop chronic critical illness (CCI). Our primary aim was to determine whether CCI sepsis survivors manifest unique blood leukocyte transcriptomes in late sepsis that differ from transcriptomes among sepsis survivors with rapid recovery. In a prospective cohort study of surgical ICU patients, genome-wide expression analysis was conducted on total leukocytes in human whole blood collected on days 1 and 14 from sepsis survivors who rapidly recovered or developed CCI, defined as ICU length of stay ≥ 14 days with persistent organ dysfunction. Both sepsis patients who developed CCI and those who rapidly recovered exhibited marked changes in genome-wide expression at day 1 which remained abnormal through day 14. Although summary changes in gene expression were similar between CCI patients and subjects who rapidly recovered, CCI patients exhibited differential expression of 185 unique genes compared with rapid recovery patients at day 14 (*p* < 0.001). The transcriptomic patterns in sepsis survivors reveal an ongoing immune dyscrasia at the level of the blood leukocyte transcriptome, consistent with persistent inflammation and immune suppression. Furthermore, the findings highlight important genes that could compose a prognostic transcriptomic metric or serve as therapeutic targets among sepsis patients that develop CCI.

## 1. Introduction

Sepsis is the leading cause of in-hospital mortality in the United States [1]. Fortunately, over the past few decades, inpatient mortality attributable to sepsis has declined [2,3]. However, this has not accelerated patient recoveries [2,4]. Advancements in the detection and treatment of surgical sepsis have led to two common clinical trajectories in those that survive: patients who rapidly recover and those who develop chronic critical illness (CCI) [2,5,6,7]. CCI is characterized by a prolonged intensive care unit (ICU) stay of 14 days or greater with ongoing organ dysfunction [6,8,9]. These patients have been determined to have persistent immunosuppression and prolonged impairment of host protective immunity [7,9]. While the majority of sepsis survivors recover, more than 40% of CCI patients die within one year, and CCI survivors exhibit a reduced quality of life [2]. CCI patients commonly manifest a pathologic endotype of low-grade chronic systemic inflammation, immunosuppression, and muscle wasting, referred to as the Persistent Inflammation, Immunosuppression, and Catabolism Syndrome (PICS) [6,10,11]. Understanding the pathobiology of CCI after sepsis and its role in long-term complications, disability, and mortality, as well as delivering precision medicine to these patients at specific time-points are considered key aspects to improving long-term sepsis outcomes [12].

Although there have been important microarray-based genome-wide expression studies that have increased our understanding of acute and subacute sepsis endotypes [13,14,15,16], studies regarding late sepsis endotypes in survivors with CCI or who rapidly recover are lacking. Our goal was to examine whether blood leukocyte transcriptomic profiles at post-sepsis days 1 and 14 are unique to clinical trajectories of CCI versus rapid recovery. We sought to identify differentially expressed genes that may be associated with the underlying immunosuppressive and inflammatory mechanisms that differentiate late sepsis phenotypes of CCI versus rapid recovery in surgical sepsis survivors. We also sought to determine whether there are transcriptomic differences between CCI patients with good versus poor hospital dispositions, as those with poor dispositions are associated with adverse one-year outcomes [2,17].

## 2. Materials and Methods

### 2.1. Study Design

The study was conducted with prior approval from the Institutional Review Board (#201702638) at the University of Florida (UF) Shands Hospital in Gainesville, FL. A total of 363 surgical intensive care unit (SICU) patients who were either admitted with or subsequently developed sepsis [18] during their hospitalization were enrolled from 1 January 2015 through to 31 December 2018, and then followed out to 1 year [6]. Following hospital discharge, patients (or their proxy) were contacted monthly by telephone concerning subsequent hospitalizations and current disposition, including mortality, which was cross-validated via the United States Social Security Death Index. Among survivors, prospective follow-up assessments were conducted at 3, 6, and 12 months after sepsis onset. These were conducted in-person at the UF Institute on Aging, at the patient’s home, or via telephone (as feasible, in that sequence).

Patients eligible for participation in the study met the following inclusion criteria: (a) admission to the surgical or trauma ICU; (b) age ≥ 18 years; (c) clinical diagnosis of sepsis, severe sepsis, or septic shock, as defined by the 2001 sepsis consensus guidelines (note the study began before the 2016 Sepsis-3 guidelines were published), and with this being the patient’s first septic episode; and, (d) entrance into our sepsis clinical management protocol [6]. Exclusion criteria eliminated patients whose baseline immunosuppression, end-stage comorbidities or severe functional disabilities would be a primary determinant of their long-term outcomes and thus confound outcome assessment, as previously described [19].

Patients in this study cohort were reclassified retrospectively with sepsis or septic shock using the Sepsis-3 definitions established by the 2016 International Sepsis Definitions Conference (Table S1) [20]. Sepsis patients were categorized as either “CCI” or “rapid recovery”, as previously described [9]. CCI was defined as an intensive care unit (ICU) length of stay (LOS) greater than or equal to 14 days with evidence of persistent organ dysfunction, measured using components of the Sequential Organ Failure Assessment (SOFA) score (i.e., cardiovascular SOFA ≥ 1, or score in any other organ system ≥ 2) [21]. Patients with an ICU length of stay (LOS) less than 14 days would also qualify for CCI if they were discharged to another hospital, a long-term acute care facility, or to a hospice and demonstrated continuing evidence of organ dysfunction at the time of discharge, as previously described [9]. Those patients experiencing death within 14 days of sepsis onset were excluded from the analyses as an early death. Any patient who did not meet criteria for CCI or death within 14 days was classified as rapid recovery.

### 2.2. Blood Collection

**Figure 1:**
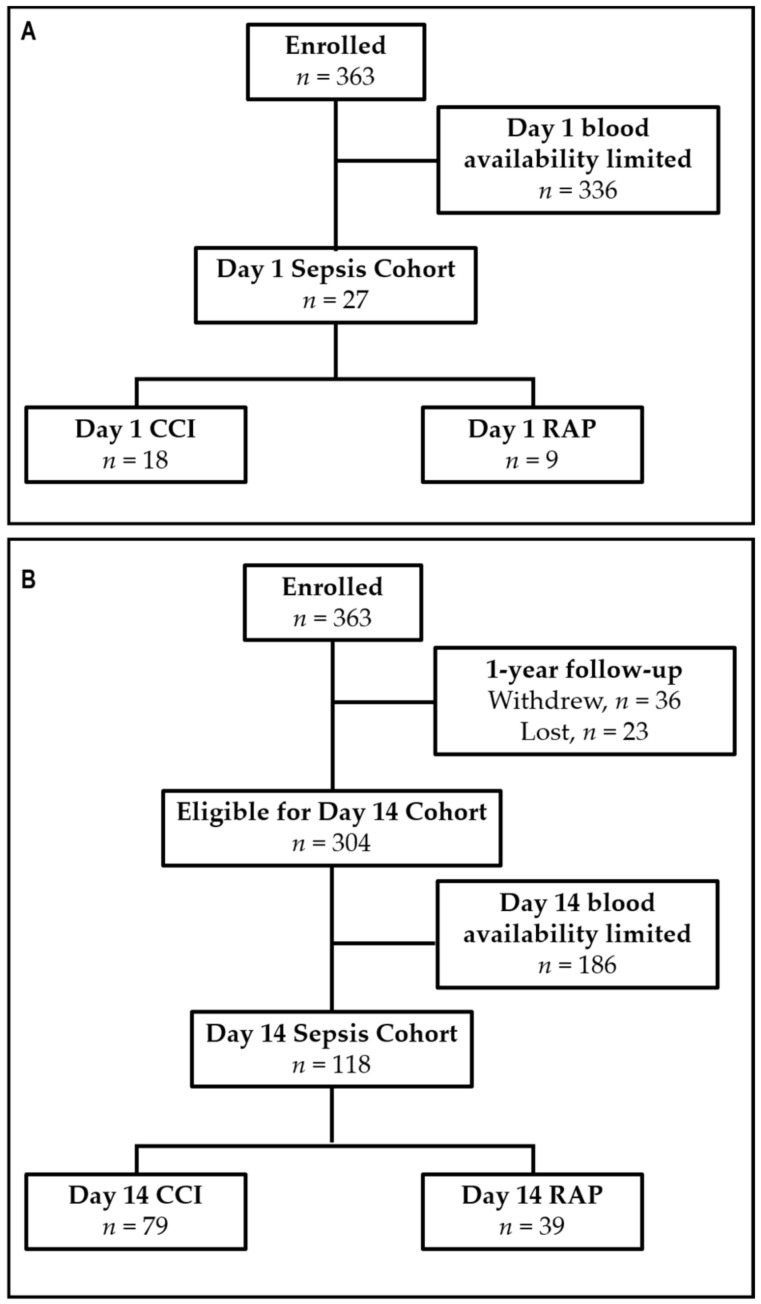
Study enrollment flowchart (**A**) for the day 1 sepsis cohort and (**B**) for day 14 sepsis cohort. CCI = Chronic Critical Illness; RAP = Rapid Recovery.

EDTA-anticoagulated whole blood was collected from sepsis patients at 1 and 14 days post-sepsis. Blood samples were stored on ice and processed within six hours. Samples for post-sepsis day 1 consisted of 18 CCI patients and age, gender, and race/ethnicity-matched to nine rapid-recovery patients. Samples for post-sepsis day 14 consisted of 79 CCI patients and 39 rapid recovery patients at discharge or day 14 (median 14 ± 2 days). The discrepancy in sample number is a result of the different blood volumes and availability required for other analyses of this patient cohort (Figure 1). Samples were also collected from 41 healthy age and sex-matched controls.

### 2.3. Gene Expression Profile and Statistical Analysis

Total blood leukocytes were isolated from whole blood. Briefly, blood was centrifuged at 500× *g* for 10 min at 4 °C and plasma removed. The red and white cell pellet was lysed with 10 volumes of Qiagen red cell lysis buffer and the process was repeated three times. Remaining white blood cells were pelleted and lysed with RLT buffer (Qiagen, Valencia, CA, USA). RNA was isolated from whole blood leukocytes using an RNeasy^®^ kit (Qiagen, Valencia, CA, USA). For amplification, 100 ngs of total cellular RNA was used. Genome-wide expression patterns were measured using HTA 2.0 GeneChips™ (Affymetrix, Santa Clara, CA, USA). BRBArray Tools^®^ (version 4.6.1, R. Simon and A. Peng-Lam, National Cancer Institute, Rockville, MD, USA)) was used to pre-process, normalize, and identify significant microarray gene expression differences. Further statistical analysis was performed using R Statistical Software (v3.5.1, R Core Team, Vienna, Austria) and SAS (v.9.3, SAS Institute Inc., Cary, NC, USA).

Gene expression differences were calculated as expression fold changes between cohorts. Significant genes were then selected using fold change (>|2|), the *p*-value, and/or the Benjamini–Hochberg multiple-test adjustment with false discovery rate (FDR) Q < 0.0001 as follows: (1) CCI and rapid recovery sepsis patients compared with age/sex-matched healthy controls at days 1 and 14 post-sepsis (genes selected with fold change and FDR); (2) direct comparison of sepsis survivors who developed CCI versus rapid recovery at days 1 and 14 post-sepsis (genes selected with *p*-value < 0.001); and (3) comparison within sepsis survivors with a good versus poor clinical disposition (genes selected with FDR Q < 0.001). Principal component analysis (PCA) was performed on subjects with CCI or who rapidly recovered at days 1 and day 14, along with controls. The data were log2-transformed and then genes were filtered for those with high variability across all samples. The elbow method was used to determine a variance cutoff of 0.4, which retained 1052 genes. The prcomp function in R was used to perform the PCA with centering and scaling.

To quantify the overall magnitude of perturbation in expression between two groups, such as CCI and rapid recovery patients, at the two time-points, a modified Distance From Reference (DFR) metric was calculated from the same subset of genes used in the PCA:

DFR = ln ∑ probe sets (e_i_ − M_i_)^2^/V_i_, (1)

where e_i_ is the patient’s expression level for probe set i, M_i_ is the mean of all controls’ expression of probe set i, and V_i_ is the variance (squared standard deviation) of all controls’ expression of probe set i. Division by the control’s variance is a rescaling method that prevents the DFR score from being dominated by genes that are inherently more variable or more highly expressed [22,23].

The primary outcome of interest for CCI patients was disposition status at discharge, determined to be either “good” or “poor” based on disposition placement. Disposition was “good” if patients were discharged to their home, with or without home care. Discharge to another inpatient hospital, hospice, long-term acute care center, specialized nursing facility, or death were considered to be “poor” dispositions. The secondary outcomes were the incidence of in-hospital mortality, mortality at 1 year, and functional status at 12 months, as indicated by the Zubrod score. Briefly, the Zubrod score is a six-point scale that measures the performance status of a patient’s ambulatory nature. The Zubrod score range is from 0 to 5, with an increasing score reflecting a worse performance status: 0, asymptomatic (fully active); 1, symptomatic but completely ambulatory (restricted in physically strenuous activity); 2, symptomatic, <50% in bed during the day (ambulatory and capable of all self-care but unable to perform any work activities); 3, symptomatic, >50% in bed, but not bedbound (capable of only limited self-care); 4, bedbound (completely disabled, incapable of any self-care); and 5, death [19].

The Kyoto Encyclopedia of Genes and Genomes (KEGG) pathways enrichment analysis of the day 14 gene set between CCI patients and those who rapidly recovered (RAP) was performed using *Enrichr* (https://maayanlab.cloud/Enrichr, accessed on 17 June 2021), with an enrichment *p*-value cutoff set to pvalueCutoff = 0.1. Results for continuous variables are reported as mean ± SD for normally distributed variables or medians (25th quartile, 75th quartile) for non-normally distributed variables. Normality was confirmed using the Shapiro–Wilk test. Student’s t-test or the nonparametric Mann–Whitney test was used to compare normal or non-normal variables, respectively, between different groups or time-points.

## 3. Results

### 3.1. Patient Demographics

There were no significant differences in sex, age, race, and BMI between CCI patients and those who rapidly recovered (Table 1). There were also no significant differences between groups with respect to primary admission diagnosis, in-hospital mortality, or the number of patient comorbidities. However, sepsis survivors with CCI demonstrated higher APACHE II scores and maximum SOFA scores at 24 h. Patients who developed CCI were more likely to have increasing sepsis severity, exhibiting a higher incidence of septic shock. Additionally, CCI patients were significantly more likely to have a “poor” disposition and worse 1-year outcomes. Of note, clinical outcomes did not appear to greatly differ between this retrospectively adjudicated Sepsis-3 patient cohort and all enrolled 365 patients (Table S1).

### 3.2. Time-Dependent Unique Leukocyte Transcriptome Pattern in CCI vs. Rapid Recovery

At the genome-wide level, sepsis produced profound changes in expression on both days 1 and 14 consistent with a ‘genomic storm’ [24,25]. CCI patients had a small, insignificant increase in the magnitude of overall genomic aberration that was evident as early as 24 h post-sepsis. This was reflected by the DFR, which is a measure of the overall summary changes in gene expression from healthy, control subjects. Specifically, the mean DFR for CCI and rapid recovery patients at day 1 were 10.63 ± 1.04 (ln expression units) and 10.49 ± 1.32, respectively, versus healthy control subjects (6.75 ± 0.57; both *p* < 0.001). However, at day 14, the overall leukocyte dyscrasia (differential transcriptomic response to sepsis), as measured by the DFR, remained essentially unchanged in both CCI (10.16 ± 1.38) and rapid recovery patients (9.97 ± 1.18) (both *p* < 0.001) consistent with a persistent aberration in gene expression, at the level of summary changes.

**Figure 2:**
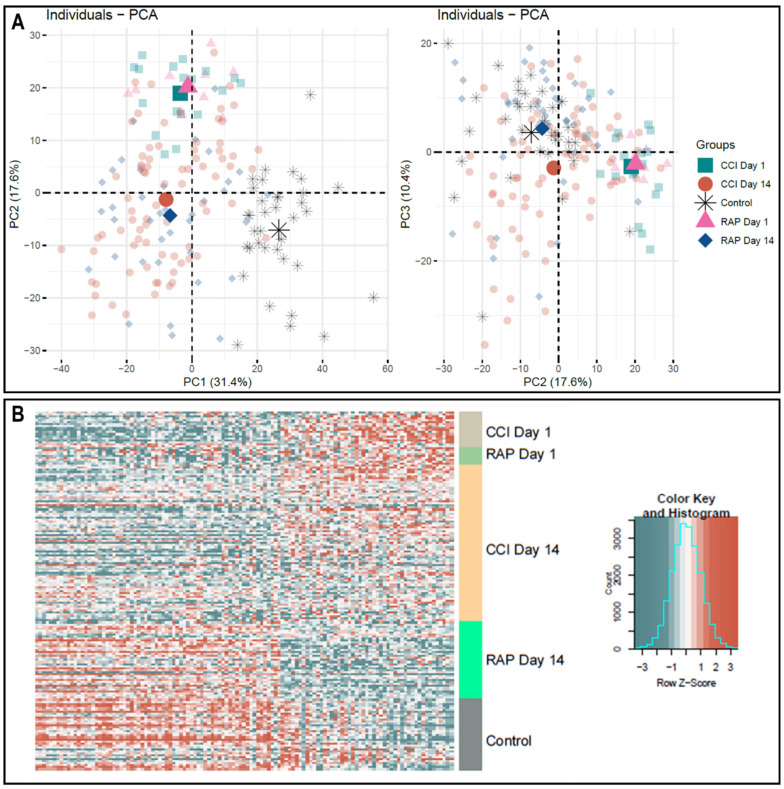
Microarray Transcriptomic Analysis of Leukocytes from Rapid Recovery and CCI patients. The genomic response of isolated total leukocyte RNA in healthy controls and sepsis patients. (**A**) Conditional principal component analysis of sepsis and healthy control leukocyte gene expression patterns from genes that had a log2 variation > 0.4 (see Materials and Methods). Each shape represents genomic expression of one group at a specific time-point. PC1 explains 31.4%, PC2, 17.6% and PC3, 10.4% of the total variation. It is in the PC2–PC3 analysis that the differences in gene expression at day 14 are most evident between patients with rapid recovery and those with CCI. (**B**) Heat map (log2) of the leukocyte gene expression patterns and variation between CCI and rapid recovery patients at day 1 and day 14 versus healthy control subjects on significant differentially expressed genes. Note the pattern of expression in rapid recovery at day 14 is closer to control than CCI at day 14, consistent with the PC2–PC3 mapping in (panel A). CCI = chronic critical illness patients, RAP = rapid recovery patients, PC = principal component.

Given the high dimensionality of gene expression data, we performed an unsupervised principal component analysis using the same set of genes in the DFR calculation. In this analysis, we observed evidence that sepsis survivors who rapidly recovered are transcriptomically closer to controls at day 14 (Figure 2A). This reflects a qualitative difference in the gene expression patterns between sepsis survivors with CCI and those who rapidly recover.

Transcriptomic analysis of CCI and rapid recovery patients revealed significant individual genomic differences between the two groups at both 1 and 14 days post-sepsis. A total of 4133 and 272 unique genes were found to be differentially expressed in leukocytes from CCI patients at 1 and 14 days post-sepsis, respectively, when compared with healthy control subjects (fold change >|2|, FDR < 0.0001). In contrast, only 1851 and 283 unique genes were found to be differentially expressed in leukocytes from rapid recovery patients at 1 and 14 days post-sepsis, respectively, when compared with healthy control subjects (fold change >|2|, FDR < 0.0001). Interestingly, CCI patients and those who rapidly recovered had 1211 and 193 common genes differentially expressed at days 1 and 14, respectively, with 100% of these genes changed in the same direction (compared with age-matched controls; Supplementary Table S2). However, the early genomic storm in CCI patients more than doubled the number of individual genes that were significantly changed compared with healthy control subjects.

Direct comparison of CCI versus rapid recovery transcriptomes revealed differential expression of 118 and 185 unique genes at 1 and 14 days post-sepsis, respectively (*p* < 0.001). Importantly, the uniquely expressed gene dataset at day 1 shared no common genes with the dataset from day 14, indicating a circulating leukocyte genomic signature that is both time- and clinical trajectory-dependent. Not surprisingly, evaluation of the differentially expressed genes in CCI versus rapid recovery revealed that expression patterns for CCI patients were not significantly more aberrant from healthy control subjects than those of rapid recovery patients during the acute phase (day 1) of sepsis (Figure 2B). However, as noted in Figure 2, panel B, the transcriptomic response from sepsis survivors who rapidly recovered appeared to more closely approximate the expression of controls at day 14.

We also utilized KEGG pathways for further analysis of our genomic data, as the use of this analysis allows greater biological insight into the functional processes likely involved in these late sepsis CCI leukocytes. Enrichment analysis identified KEGG pathways significant for T-helper and hematopoietic cell differentiation, antigen-processing and presentation, and an intestinal immune network for IgA production in CCI patients at day 14 (Table S2).

### 3.3. Unique Leukocyte Transcriptomic Pattern in Patients with Adverse Clinical Outcomes

Consistent with our previous reports [2,9], sepsis survivors with CCI in this study cohort were more likely to have a higher percentage of patients with poor discharge dispositions (77% of CCI vs. 33% of rapid recovery, *p* < 0.001; Table 1). Additional comparison of day 14 post-sepsis CCI patients with a good disposition (*n* = 18) to those with poor disposition (*n* = 61) demonstrated 306 differentially expressed genes (*p* < 0.001; Table S3). CCI patients with good disposition had decreased expression of all these genes when compared with the CCI patients with poor disposition. Additional comparison at day 14 post-sepsis of all sepsis patients with a good (*n* = 44) compared with a poor (*n* = 74) discharge disposition demonstrated 620 differentially expressed genes (*p* < 0.001; Table S3). Again, sepsis survivors with a good disposition had decreased expression of all the differentially expressed genes when compared with those with poor disposition. Many of these genes have previously been noted to be important in immune cell and stem cell function, such as *BLK, BAG6, FOXO4,* and *ERF* (Table 2) [26,27,28]. However, the upregulation of these genes in poor disposition seems to indicate a persistent unwarranted inflammatory response at day 14 in patients with dismal outcomes after sepsis. Importantly, these latter two findings suggest that at the genome-wide expression level (DFR), the return to baseline was similar between CCI and rapid recovery cohorts, and this was not the case for selected genes involved in host protective immunity (Table S3).

## 4. Discussion

This study is the first to report late blood leukocyte transcriptomic differences between surgical sepsis survivors who developed CCI or rapidly recovered. We postulated that CCI is itself a unique phenotype in sepsis survivors that differs substantially from the early genomic storm and the patterns seen in patients who rapidly recover. Our results confirm that there are unique transcriptomic patterns at day 1 and day 14 in sepsis survivors who develop CCI when compared with patients who rapidly recover. Additionally, we have demonstrated that there are further transcriptomic differences within the septic CCI cohort of patients that are associated with good versus poor hospital dispositions after surgical sepsis. Finally, our work supports the conclusion that patients who exhibit CCI have persistent low-grade inflammation and immunosuppression, which is known to contribute to poor outcomes [8,29,30,31] and represents a portion of the pathobiology of the Persistent Inflammation, Immunosuppression and Catabolism Syndrome (PICS).

Similar to previous studies, our study supports the hypothesis that immune dysregulation characterizes sepsis survivors in general, and in particular those who develop CCI. As noted in other sepsis cohorts, the transcriptional changes in inflammatory genes, both early and late after sepsis, for CCI and rapid recovery patients are highly variable [32,33,34,35,36,37,38,39]. Neither early nor late sepsis transcriptomic patterns identify a distinctive pro-inflammatory or immunosuppressive phase. Endotyping sepsis survivors into broad classes as either ‘proinflammatory’ or ‘immunosuppressive’ is overly simplistic. Rather, there is an aberrance of many inflammatory and immunosuppressive genes simultaneously, suggesting a more global immune dysregulation, consistent with the PICS endotype. KEGG enrichment analysis demonstrated that the differentially expressed genes from total blood leukocytes between CCI and rapidly recovered sepsis patients are involved in many important immunologic pathways, such has T-helper cell differentiation. By day 14, the number of genes whose expression differed between the sepsis subgroups and healthy controls were roughly the same as the number of genes whose expression differed between CCI and rapid recovery patients. This suggests that although both groups showed a genome-wide summary pattern of return towards baseline, the individual genes whose expression remained aberrant were different between the two sepsis outcome groups. Such a finding suggests that the late immunological endotype associated with CCI is characterized by both inflammation and immune suppression, rather than one or the other.

Previous studies in other disease states have suggested that transcriptomic analysis of circulating leukocytes could be used to identify clinical outcomes [40,41,42,43]. Historically, transcriptomic metrics have been used to differentiate infectious versus noninfectious causes in early critical illness [13,15,16,44]. Although our study confirms that transcriptomic patterns between CCI and rapid recovery patients differ in both early and late sepsis, transcriptomic prediction of long-term outcomes in the first 24 h of sepsis may not be possible for all CCI phenotypes, as there are multiple other factors that impact the trajectory of sepsis [45]. However, our findings suggest that the unique pattern of 185 genes differentially expressed at 14 days may prove useful for identifying those CCI patients who have increased risk of 1-year mortality. Not only could a metric be crafted as a prognostic tool, but the differential expression could be used for more focused immunomodulation therapies to improve overall outcomes to sepsis. In addition, other factors can potentially be utilized alone or in combination with the transcriptomics to successfully conduct personalized/precision medicine in this patient population [46,47].

The main limitation of this study was that it was performed at a single institution in a limited number of surgical sepsis patients. Additionally, we were only able to analyze leukocyte transcriptome patterns at 1 and 14 days after sepsis. We believe these two data-points are not completely adequate to provide an appropriate dynamic time-course analysis of genomic expression. Future transcriptomic analysis at greater than two time-points is warranted to analyze time-dependent genomic expression patterns of sepsis in sepsis survivors. Finally, the transcriptomics in this study represent all circulating leukocytes, yet individual immune cell populations each play specific and unique roles in the development and subsequent pathology of sepsis. Additionally, differences in immune cell distribution may also play a role in sepsis clinical trajectory and outcomes. In our study, measurements reflecting immune cell distribution at day 14 (white blood cell count, lymphocyte count and neutrophil counts) were significantly different between CCI and rapid recovery cohorts (*p* < 0.01, data not shown). Although this study provides insights into specific transcriptomic changes that may underlie the pathobiologic syndrome of low-grade chronic systemic inflammation, immunosuppression, and muscle wasting seen in CCI patients after sepsis, studies are currently underway using novel technology, such as single-cell RNAseq (scRNAseq), Cellular Indexing of Transcriptomes and Epitopes by Sequencing (CITE-seq), and Assay for Transposase Accessible Chromatin using sequencing (ATAC-seq) to better comprehend how each cell type may contribute to clinical trajectories of sepsis. However, the work presented in this manuscript highlights a number of important genes and cell populations that warrant further investigation for targeted therapy in those sepsis patients that have CCI and adverse outcomes.

## 5. Conclusions

Surgical sepsis patients who develop CCI have a unique circulating leukocyte transcriptomic pattern at both 1 and 14 days post-sepsis compared with sepsis survivors who rapidly recover. Our data support the hypothesis that CCI represents a unique late sepsis phenotype characterized by a pattern of dysfunctional and simultaneous inflammation and immunosuppression. In addition, the gene expression profile of CCI patients with good versus poor disposition differs. These findings could help prognosticate patient outcomes, as well as determine which CCI patients may benefit from targeted immunotherapies. Since some CCI patients do recover, further analysis may allow the application of precision medicine after sepsis, that is, prediction of which patients require immunomodulation, the type of therapy required, and the timing of treatment.

## Figures and Tables

**Table 1:** Characteristics of Day 14 Sepsis Cohort with * Univariate Analysis between CCI and rapid recovery subjects.

	Overall (*n* = 118)	CCI (*n* = 79)	RAP (*n* = 39)	*p*-Value
Demographics				
Male, *n* (%)	67 (56.8)	48 (60.8)	19 (48.7)	0.2401
Age in years, mean (SD)	60.3 (15)	62.1 (13.4)	56.5 (17.3)	0.101
Age ≥ 65, *n* (%)	49 (41.5)	34 (43)	15 (38.5)	0.6941
Race, *n* (%)				0.3041
Caucasian	104 (88.1)	71 (89.9)	33 (84.6)	
African American	11 (9.3)	7 (8.9)	4 (10.3)	
Asian	1 (0.8)	0 (0)	1 (2.6)	
Other	2 (1.6)	1 (1.3)	1 (2.6)	
BMI, median (25th, 75th)	29.5 (24.4, 38)	29.5 (24.7, 39.2)	28.7 (24.1, 37.3)	0.6288
Charlson comorbidity index, median (25th, 75th)	3 (2, 5)	3 (2, 5)	3 (0, 4)	0.0834
APACHE II, median (25th, 75th)	20 (14, 25)	22 (16, 26)	16 (11, 22)	0.0029
Inter-facility hospital transfer, *n* (%)	55 (46.6)	41 (51.9)	14 (35.9)	0.119
Sepsis severity by Sepsis 3 criteria, *n* (%)				0.1401
Sepsis	83 (70.3)	52 (65.8)	31 (79.5)	
Septic shock	35 (29.7)	27 (34.2)	8 (20.5)	
Primary Sepsis Diagnosis, *n* (%)				0.36
CLABSI/Bacteremia	2 (1.7)	1 (1.3)	1 (2.6)	
De Novo Intra-Abdominal Infection	27 (22.9)	20 (25.3)	7 (17.9)	
Necrotizing Soft Tissue Infection	15 (12.7)	6 (7.6)	9 (23.1)	
Pneumonia	26 (22)	19 (24.1)	7 (17.9)	
Surgical Site Infection	34 (28.8)	23 (29.1)	11 (28.2)	
Urosepsis	6 (5.1)	4 (5.1)	2 (5.1)	
Other	8 (6.8)	6 (7.6)	2 (5.1)	
Creatinine at sepsis onset, median (25th, 75th)	1.1 (0.7, 1.7)	1.1 (0.8, 2)	1.1 (0.6, 1.2)	0.1773
ALC at sepsis onset, median (25th, 75th)	0.3 (0, 0.6)	0.3 (0, 0.6)	0.2 (0, 0.6)	0.6492
Lactate at sepsis onset, median (25th, 75th)	1.7 (1.1, 2.7)	1.8 (1.2, 2.8)	1.5 (1.1, 2.7)	0.6106
Inpatient outcomes				
In-hospital mortality, *n* (%)	6 (5.1)	6 (7.6)	0 (0)	0.1761
ICU Length of Stay (LOS), median (25th, 75th)	19 (11, 28)	24 (18, 39)	9 (5, 12)	<0.0001
Hospital LOS, median (25th, 75th)	28 (21, 38)	32 (24, 48)	21 (17, 30)	<0.0001
Max SOFA score 24 h, median (25th, 75th)	9 (7, 12)	10 (9, 13)	7 (5, 9)	<0.0001
Multiple Organ Failure incidence, *n* (%)	74 (62.7)	60 (75.9)	14 (35.9)	<0.0001
Discharge disposition, *n* (%)				
“Good” disposition	44 (37.3)	18 (22.8)	26 (66.7)	<0.0001
Home	7 (5.9)	1 (1.3)	6 (15.4)	
Home healthcare services	26 (22)	9 (11.4)	17 (43.6)	
Rehab	11 (9.3)	8 (10.1)	3 (7.7)	
“Poor” disposition	74 (62.7)	61 (77.2)	13 (33.3)	<0.0001
Long Term Acute Care facility	34 (28.8)	34 (43)	0 (0)	
Skilled Nursing facility	20 (16.9)	8 (10.1)	12 (30.8)	
Another Hospital	9 (7.6)	8 (10.1)	1 (2.6)	
Hospice	5 (4.2)	5 (6.3)	0 (0)	
Death	6 (5.1)	6 (7.6)	0 (0)	
30-day mortality, *n* (%)	8 (6.8)	7 (8.9)	1 (2.6)	0.2679
12-month mortality, *n* (%)	35 (29.7)	33 (41.8)	2 (5.1)	<0.0001
Zubrod at 12 months, median (25th, 75th)	3 (1, 5)	4 (2, 5)	1 (1, 3)	<0.0001

ALC = Absolute Lymphocyte Count; BMI = Body Mass Index; CCI = Chronic Critical illness; RAP = Rapid Recovery; ICU = Intensive Care Unit; SOFA = Sequential Organ Failure Assessment Score.

**Table 2:** Select Genes Found to be Significantly Altered at Day 14 Post-Sepsis in Sepsis Survivors who Developed CCI with Good versus Poor Outcomes.

Genes	Function
*ATG12*	Promotes autophagy
*BAG6*	Antigen degradation and immune cell function and response
*BLK*	B-cell development and signaling
*EHD1*	IL-2 secretion and T-cell proliferation
*ERF*	Hematopoietic stem cell differentiation
*FOXO4*	Quiescence and maintenance of hematopoietic stem cells
*NACC1*	Stem cell self-renewal and maintenance
*SLC7A5*	T-cell differentiation

## Data Availability

The data presented in this study are available on request from the corresponding author.

## Supplementary Materials

The following are available online at https://www.mdpi.com/article/10.3390/jcm10153211/s1, Table S1: Characteristics of All Enrolled Sepsis Patients in Comparison to Selected Sepsis Cohort. Table S2: List of significant gene probe sets (FDR < 0.01 and Fold Change > |2|) in chronic critical illness (CCI) sepsis patients and rapid recovery patients at day 14 versus healthy controls. Table S3: List of significant gene probe sets (*p* < 0.001) in good vs. poor disposition within all sepsis patients and chronic critical illness (CCI) sepsis patients at day 14.
