# Supplementary material for: Chronic Critical Illness Elicits a Unique Circulating Leukocyte Transcriptome in Sepsis Survivors
Source: J Clin Med. 2021 Jul 21;10(15):3211. doi: 10.3390/jcm10153211 (PMC8348105; doi:10.3390/jcm10153211)
Supplement: Supplementary file 1 [file jcm-10-03211-s001.zip › Table S1.pdf]

Table S1. Characteristics of All Enrolled Sepsis Patients in Comparison to Selected Sepsis Cohort.

	**Study Cohort (*n* = 118)**	**Total Enrolled (*n* = 363)**
Male, *n* (%)	67 (56.8)	196 (54)
Age in years, mean (SD)	60.3 (15)	59 (15.5)
Age ≥ 65, *n* (%)	49 (41.5)	145 (39.9)
Race, *n* (%)		
Caucasian	104 (88.1)	324 (89.3)
African American	11 (9.3)	35 (9.6)
American Indian	1 (0.8)	1 (0.3)
Asian	1 (0.8)	1 (0.3)
Other	1 (0.8)	1 (0.3)
Unknown	0 (0)	1 (0.3)
BMI, median (25th, 75th)	29.5 (24.4, 38)	29.5 (24.9, 36.9)
Charlson comorbidity index, median (25th, 75th)	3 (2, 5)	3 (1, 5)
APACHE II, median (25th, 75th)	20 (14, 25)	17 (11, 23)
Inter-facility hospital transfer, *n* (%)	55 (46.6)	152 (41.9)
Sepsis severity by Sepsis 2 criteria, *n* (%)		
Sepsis	20 (16.9)	110 (30.3)
Severe sepsis	57 (48.3)	156 (43)
Septic shock	41 (34.7)	97 (26.7)
Sepsis severity by Sepsis 3 criteria, *n* (%)		
Does not qualify	0 (0)	20 (5.5)
Sepsis	83 (70.3)	255 (70.2)
Septic shock	35 (29.7)	88 (24.2)
Primary Sepsis Diagnosis, *n* (%)		
CLABSI/Bacteremia	2 (1.7)	8 (2.2)
De Novo Intra-Abdominal Infection	27 (22.9)	105 (28.9)
Necrotizing Soft Tissue Infection	15 (12.7)	43 (11.8)
Pneumonia	26 (22)	58 (16)
Surgical Site Infection	34 (28.8)	82 (22.6)
Urosepsis	6 (5.1)	41 (11.3)
Other	8 (6.8)	26 (7.2)
Creatinine at sepsis onset, median (25th, 75th)	1.1 (0.7, 1.7)	1.1 (0.7, 1.7)
ALC at sepsis onset, median (25th, 75th)	0.3 (0, 0.6)	0.6 (0.3, 0.9)
Lactate at sepsis onset, median (25th, 75th)	1.7 (1.1, 2.7)	1.7 (1.2, 2.8)
In-hospital mortality, *n* (%)	6 (5.1)	28 (7.7)
ICU Length of Stay (LOS), median (25th, 75th)	19 (11, 28)	7 (3, 17)
Hospital LOS, median (25th, 75th)	28 (21, 38)	15 (8, 26)
Maximum SOFA score 24 hours, median (25th, 75th)	9 (7, 12)	7 (5, 10)

Multiple Organ Failure incidence, *n* (%)	74 (62.7)	153 (42.3)
Clinical trajectory, *n* (%)		
Early Death	0 (0)	14 (3.9)
CCI	79 (66.9)	124 (34.2)
RAP	39 (33.1)	225 (61.9)
Discharge disposition, *n* (%)		
"Good" disposition	44 (37.3)	207 (57)
Home	7 (5.9)	72 (19.8)
Home healthcare services	26 (22)	109 (30)
Rehab	11 (9.3)	26 (7.2)
"Poor" disposition	74 (62.7)	156 (43)
Long Term Acute Care facility	34 (28.8)	54 (14.9)
Skilled Nursing facility	20 (16.9)	53 (14.6)
Another Hospital	9 (7.6)	13 (3.6)
Hospice	5 (4.2)	8 (2.2)
Death	6 (5.1)	28 (7.7)
30-day mortality, *n* (%)	8 (6.8)	33 (9.4)
12-month mortality, *n* (%)	35 (29.7)	76 (23.2)
Zubrod at 12 months, median (25th, 75th)	3 (1, 5)	1 (1, 4.5)

CCI = Chronic Critical Illness; RAP = Rapid Recovery; BMI = Body Mass Index; ALC = Absolute Lymphocyte Count; ICU = Intensive Care Unit;, SOFA = Sequential Organ Failure Assessment Score.
